# Tomographic Assessment of Bone Regeneration in Osteochondral Lesion Treated with Various Biomaterials in a Sheep Model Study

**DOI:** 10.3390/jfb16040120

**Published:** 2025-04-01

**Authors:** Taulant Goga, Bledar Goxha, Alberto Maria Crovace, Mario Cinone, Luca Lacitignola, Marta Guadalupi, Erinda Lika

**Affiliations:** 1 Department of Precision and Regenerative Medicine and Ionian Area, University of Bari “ALDO MORO”, 70010 Bari, Italy; bgoxha@ubt.edu.al (B.G.); mario.cinone@uniba.it (M.C.); luca.lacitignola@uniba.it (L.L.); marta.guadalupi@uniba.it (M.G.); 2 Faculty of Veterinary Medicine, Agricultural University of Tirana, 1025 Tirana, Albania; elika@ubt.edu.al; 3 Department of Veterinary Medicine, University of Sassari, 07100 Sassari, Italy; acrovace@uniss.it

**Keywords:** osteochondral lesion, tomography, scaffolds, hounsfield units, ROI value

## Abstract

Osteochondral defects, involving both articular cartilage and subchondral bone, pose significant challenges to joint function and health due to the lack of spontaneous healing and the risk of long-term degenerative diseases like osteoarthritis. Biomaterials have emerged as important components in the development of scaffolds, providing structural support that facilitates tissue growth, integration, and regeneration. This study aims to demonstrate the effectiveness of a tomographic assessment method for optimizing the evaluation of osteochondral regeneration, particularly using Hounsfield units, to enable the evaluation of scaffold integration and tissue regeneration. The sheep model was selected as a model study. Two distinct configurations of biomaterials were utilized in this study: Honey (HMG—Mg doped hydroxyapatite; HWS—wollastonite–hydroxyapatite) and Bi-layer (BWS—wollastonite–hydroxyapatite). The HMG scaffold demonstrated superior integration, reparative tissue quality, and regeneration potential compared to the HWS, BWS, and CTRL groups. The findings underscore the significance of CT assessment as a preliminary method for evaluating hard tissue, such as bone, employing Hounsfield units. Statistical evaluations validated the significant differences in performance, particularly favoring the HMG group. The results of this study underscore the importance of tomographic assessment in evaluation of osteochondral regeneration.

## 1. Introduction

Osteochondral defects represent lesions involving both the articular cartilage and the underlying subchondral bone, both of which are integral to joint function and overall health. These defects are of particular concern, as the osteochondral unit plays a crucial role in joint lubrication and the transmission of mechanical loads to the bones during movement [1]. Osteochondral defects are a common problem in both medical and veterinary practice, with difficult resolution because current techniques have significant limitations, particularly in terms of cartilaginous tissue regeneration [2,3]. Unfortunately, the osteochondral unit does not have the ability to heal spontaneously, making osteochondral lesions an important risk factor for the development of long-term degenerative joint diseases such as osteoarthritis [1]. The management of osteochondral lesions presents significant challenges due to the complex nature of the tissues involved. Articular cartilage and subchondral bone possess distinct biological and mechanical properties, yet they must operate as an integrated unit. Damage to the subchondral bone often impairs the repair process, leading to the formation of fibrous scar tissue rather than functional cartilage [4]. The inadequate healing response highlights the necessity for effective treatment strategies that target both the cartilage and subchondral bone. Over the years, a range of therapeutic approaches have been explored, spanning from non-operative methods to advanced surgical interventions. Nonoperative treatments have showed limited success, with an average success rate of approximately 45%, less than surgical procedures such as excision, curettage, and drilling, which have demonstrated an 86% success rate [5]. Recent advancements in tissue engineering such as the use of scaffold-free allogeneic constructs from adipose-derived stem cells have demonstrated significant histological healing in animal models [6]. Additionally, the development of biphasic and triphasic scaffolds aims to mimic the natural osteochondral unit, promoting the regeneration of both cartilage and subchondral bone [7,8]. Despite these advancements, the clinical translation of these strategies remains limited, as the risk of immune rejection, inflammatory reactions, or foreign body responses can affect the integration and long-term success of implanted biomaterials. Biomaterials are essential, particularly for the replacement of diseased tissues and organs and for the treatment of chronic illnesses that aim to return the body condition to its normal state of health [9]. Accurate assessment of osteochondral biomaterials is essential to ensure successful tissue regeneration and scaffold integration. Typically, biomaterials serve as a scaffold to offer the necessary structural support for tissue growth as well as cell adherence [10]. Several scaffolds have been developed for osteochondral lesions [11] combining cell-free strategies and the use of different layers to mimic the bone–cartilage structure [12,13,14]. It is worth noting that the design of bone and osteochondral scaffolds must enable hierarchical composite porous structures to achieve the desired mechanical function and mass transport, and to produce these structures within arbitrary and complex three-dimensional (3D) anatomical shapes [15]. In this study, two types of scaffolds configurations are used; the first configuration, called “Bi-layer”, a highly porous ceramic scaffold, was circumferentially surrounded by a thin layer of collagen and was characterized by a stable pre-integration between the osteo (ceramic) and chondral (collagen) components, easy surgical handling, and compliance at the implant site. In the second configuration, called “Honey”, highly porous hydroxyapatite columns were inserted into the cylindrical collagen scaffold. An osteochondral lesion of the knee, which is a localized anomaly of the subchondral marrow, subchondral bone, and articular cartilage, can be a symptom of several pathologic diseases [16]. Macroscopic assessment methods enable a visual and structural evaluation of scaffold morphology and integration on a surface level. In contrast, tomographic techniques, especially those involving Hounsfield units (HUs) in CT imaging, provide insight into internal structural changes, bone density, and scaffold integration with surrounding tissues, making it possible to monitor tissue remodeling and regeneration over time. HUs provide a way to differentiate between soft tissues (like cartilage) and bone. While cartilage is not as clearly visible on CT scans compared to MRI, changes in the subchondral bone beneath the cartilage, which is crucial in osteochondral lesions, are easily identifiable on CT using HUs [17]. HU measurements can be used to monitor the progression or healing of osteochondral lesions over time. During the healing process, changes in the HU values can indicate new bone formation or ongoing bone remodeling, which are signs of recovery. Conversely, if there is a continued decrease in bone density, it might suggest worsening or incomplete healing [17,18]. Evaluating osteochondral scaffolds via HUs is crucial for assessing the quality and success of scaffolds used in cartilage and bone repair. The use of HU from CT scans in this evaluation allows for a quantitative and objective assessment of scaffold integration, bone regeneration, and overall healing of the osteochondral lesion. CT imaging with HU measurements allows for the quantification of bone density changes within and around the scaffold over time [19]. This study aims to evaluate the healing of large osteochondral defects using scaffolds with different biomaterials in osteochondral structures during regenerative processes through the use of HUs in tomographic evaluation in a 3-month period.

## 2. Materials and Methods

### 2.1. Ethical Approval

This study was conducted in the Section of Veterinary Clinics and Animal Production of the Department of Precision and Regenerative Medicine and Ionian Area of the University of Bari. The study was performed in accordance with the guidelines and approval from the Italian Ministry of Health and in strict adherence to the recommendations outlined in the National Institutes of Health Guide for the Care and Use of Laboratory Animals (D.L. 26/2014).

### 2.2. Animals

Sheep selected for the experimental treatment were identified and acclimatized for 30 days. The study population consisted of 24 adult female sheep of the Bergamasca breed, aged six years and weighing 50 ± 4 kg, all in good health. There are some scientific reasons behind selecting female sheep to be included in this study compared to males; they are calmer, safer, easier to handle due to anatomical and physiological characteristics that closely resemble those of humans, and exhibit more predictable behavior, physiological responses, and lower levels of aggression [20,21]. Prior to the initiation of the experimental procedures, ultrasound examinations were conducted to ascertain the absence of pregnancy, and the sheep were treated with one dose of anthelmintic drug Ivermectin intramuscular administration (Ivomec, Boehringer Ingelheim; 1 mL/50 kg body weight) to fight any gastrointestinal parasites. Furthermore, preoperative orthopedic evaluations and X-rays were conducted to exclude the presence of preexisting orthopedic lesions and to assess the condition of the condylar region of the right femur.

### 2.3. Scaffolds

**Figure 1:**
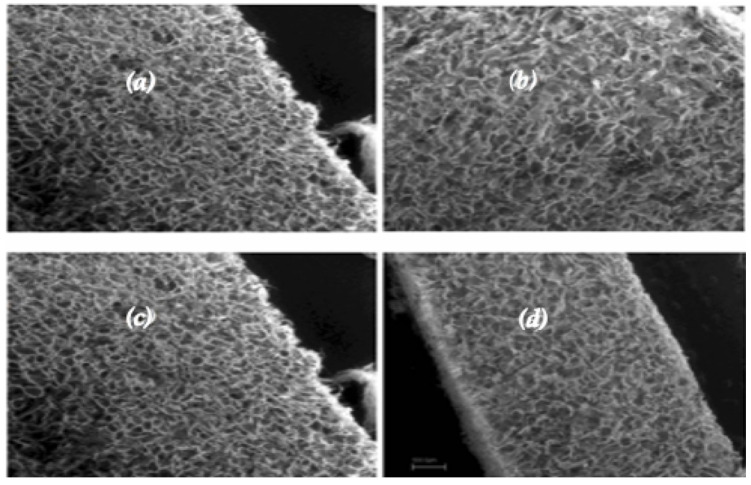
SEM images of the collagen scaffold before cell seeding. Longitudinal (**a**) and transversal (**b**) section of a FAST sample; FAST (**c**) and SLOW (**d**) samples in a longitudinal section. From the SEM images acquired on the sample freeze-dried at two different freezing rates, it is possible to notice that when the freezing velocity decreased, the pore size increased.

In this study, two different configurations of biomaterials, Honey and Bi-layer, with a scaffold size of 11 mm in diameter and 9.0 mm in height, were used. Both the biomaterials were synthesized with the foam replication method [22,23,24,25,26,27] and composed of Mg doped hydroxyapatite (Mg-HA, HMG group) or wollastonite–hydroxyapatite (WS/HA, HWS group) mixtures. Mg-HA was prepared by replacing a 10% molar of calcium ions, keeping the (Ca + Mg)/P = 1.67. WS/HA scaffold was prepared by sintering WS/HA mixtures powders in a 1/1 ratio. The Bi-layer scaffolds were then produced accordingly to the procedure previously reported [28] and the bioceramic pillars were embedded in the collagen type I layer [29,30] to form the biphasic Honey scaffolds [31]. The organic component, made of collagen, was fabricated by freeze-drying a collagen solution (2% wt/v) following a previously reported protocol [25]. A hydroxyapatite macrochanneled porous scaffold was produced by polymer sponge templating method using a reactive submicron powder synthesized through a hydroxide precipitation sol–gel route. All biphasic substitutes were sterilized in an oven under a vacuum at 160 °C for 2–4 h before implantation [24]. Bi-layer and Honey scaffolds have been characterized in terms of morphology by scanning electron microscopy (SEM Zeiss Evo40, Jena, Germany), as shown in Figure 1. The mechanical properties of the scaffolds were evaluated using compression tests. The test was performed using a standard testing machine (Lloyd LR5K instrument, Ametek, FL, USA) equipped with a 1 kN load cell, compressed at a crosshead speed of 0.5 mm/min and the maximum stress calculated. The mechanical properties of the scaffolds were evaluated by uniaxial compression tests, revealing an average stress at a failure of 0.51 MPa, which is higher than or significantly higher than previously reported values [32,33,34,35]. The morphology and mechanical properties of the scaffolds were analyzed by scanning electron microscopy and by compression tests in PBS for up to 18% of the sample un-stressed length in six equal steps at 3 min intervals (strain rate = 0.2%/s, loading cell of 10 N). The ceramic phase composition by X-ray diffraction (XRD) was evaluated by D-maxd/Ultima diffractometer (Rigaku, Tokyo, Japan) using CuKa radiation (λ = 1.5418 A°) in the step scanning mode recorded in the 2θ range of 20–60°, with a step size of 0.02° and step duration of 0.5 s. These results suggest that the scaffold possesses sufficient mechanical strength for bone tissue engineering applications, particularly in non-load-bearing bone defects. Additionally, the open and highly interconnected porosity, along with the absence of cracks, confirms the good structural integrity of the scaffolds, which is crucial for cellular infiltration and nutrient diffusion.

### 2.4. Anesthesia and Surgical Procedures

**Figure 2:**
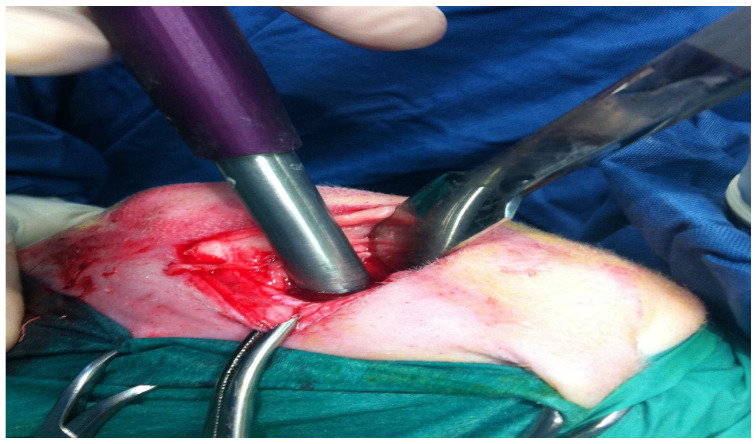
Osteochondral lesion practiced with core millimeter punch inserted to a depth of 9 mm.

All surgeries were performed by the same surgical team under aseptic conditions, utilizing both general and spinal anesthesia to minimize pain and discomfort. The sheep were fasted for 24 h prior to surgery, the back part of the earlobe was shaved and disinfected, an intravenous catheter (20G) was placed in the auricular vein, and fluid therapy was performed with Ringer’s lactate (10 mL/kg/h) adjusted as necessary according to the condition of the sheep. Sedation was induced with midazolam (Midazolam 5 mg/mL, IBI, Aprilia, Italy; 0.4 mg/kg IV), and the sheep were treated with flunixin meglumine (Flunixin 50 mg/mL, Norbrook, Agri Laboratories, Newry, UK, 1 mg/kg IV) for pain management. Once sedation was achieved, the sheep were placed in right lateral decubitus for spinal anesthesia at the level of the L6–S1 intervertebral space. The lumbosacral area was shaved and sterilized, and spinal anesthesia was administered via subarachnoid injection of lidocaine (Lidocaina 2%, Esteve Veterinaria SPA, Milano, Italy, 2 mg/kg) and buprenorphine (Buprenodale 0.3 mg/mL, Dechra, Italy, 300 mcg). During the procedure, oxygen was provided through a nasal catheter, and propofol (Propofol 1%, Fresenius Kabi, Bad Homburg, Germany, 1 mg/kg IV) was administered if the sheep exhibited signs of distress. Anesthetic monitoring was performed every five minutes, with data recorded on heart rate, respiratory rate, pulse oximetry, capnometry, capnography, systolic and diastolic arterial pressure, peak airway pressure, and mean airway pressure. The 24 sheep were positioned in dorsal recumbency with the right knee in flexion. The skin in the surgical area was shaved and disinfected. Following the application of surgical drapes, a longitudinal incision of approximately 5–7 cm was made along the medial aspect of the right knee, corresponding to the projection area of the previously identified medial condyle. A capsulotomy was performed, and in cases of hypertrophy, partial removal of the synovium was conducted to improve exposure of the condyle. The knee was then hyperflexed to better visualize the right condyle, and an osteochondral defect was created in the load-bearing region of the condyle using custom-made instruments (Core Punch, Sleeve, Hand Drill), where scaffolds were implanted using a press-fit technique, as shown in Figure 2. The lesion measured 11 mm in diameter and 9 mm in depth, matching the size of the scaffold to be implanted; the defect was clean and bordered by healthy cartilage.

The sheep were divided into four groups. The first group (n = 6) received implants of hydroxyapatite–wollastonite and collagen scaffolds (HWS), the second group (n = 6) received hydroxyapatite–magnesium and collagen scaffolds (HMS), and the third group (n = 6) received bifacial wollastonite and collagen scaffolds (BWS). The remaining six sheep served as the control group. In the control group, six lesions were left untreated (CTRL). The capsule and the subcutaneous tissue were closed with absorbable sutures and the skin was closed with non-absorbable sutures. A waterproof medication was put on the lesions; the animals were free to move without any bandage or immobilization tool. Postoperative X-ray controls were practiced in all treated animals to evaluate the position of the scaffolds. After the surgery, the sheep were monitored until they recovered from the quadrupedal station. All the animals treated were able to stand on their four legs and to bear the load on the operated limb 1–3 h after the surgery. All the operated animals received benzylpenicillin procaine (Izotricillina 100 mL, IZO SRL, Italy, 10 mL of a 200,000 UI/mL solution) and flunixin (Flunixin 50 mg/mL, Norbrook, Agri Laboratories, Newry, UK, 1 mg/kg) every 24 h for seven days. All sheep were monitored daily to detect any alteration of the clinical condition (food intake, weight loss, urine and feces production, rectal temperature, lameness, and behavioral changes). After three months, all the animals were euthanized with the administration of dexmedetomidine (Dexdomitor; Zoetis, Kalamazoo, MI, USA) at 5 μg/kg IV 5 min prior to the injection of a pentobarbital (Beuthanasia-D Special; Merck, Madison, NJ, USA) overdose given IV [36]. The right limbs were then harvested without opening the knee and further analyzed, starting with CT evaluation.

### 2.5. CT Evaluation

**Figure 3:**
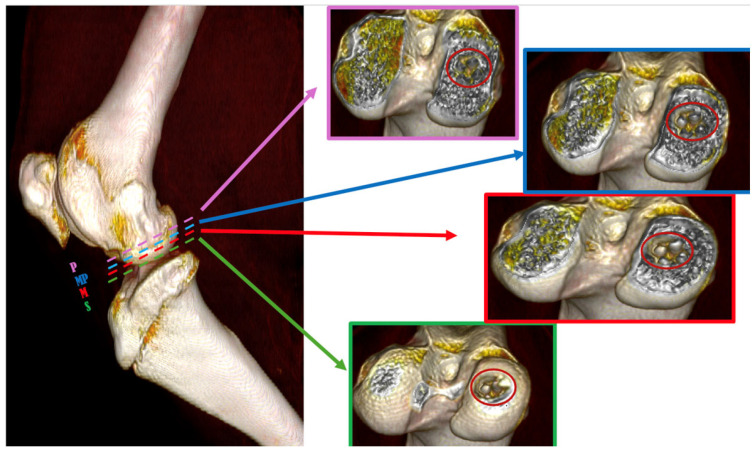
Three-dimensional reconstruction of the section under CT assessment reveals four evaluation positions of biomaterials inside the osteochondral part of the medial femoral condyle; (S) → green dashed line represents superficial position, (M) → red dashed line represents medium position, (MP) → blue dashed line represents medio-profonde position and (P) → purple dashed line represents profonde position.

The right limb was positioned in a craniocaudal direction and scanned from the distal femoral diaphysis to the proximal tibial diaphysis using a GE BrightSpeed 16-slice CT machine. The following protocol was utilized: helical mode, slice thickness 1.25 mm, 120 kVp, 200 mA, pitch 0.93, convolution kernel “bone plus”. The HOROS application was used to evaluate the CT images, and measurements were taken in four positions of the scaffold implemented in the osteochondral structure (see Figure 3). A 3D reconstruction from the distal femoral diaphysis to the proximal tibial diaphysis was performed. The ROI metrics evaluation was conducted by delineating the area of the scaffold implementation using the following commands: Closed Polygon, CT-Bone, No CLUT, Linear Table, and others. The application subsequently displayed all pertinent values in Hounsfield units, including area (cm^2^), length (cm), minimum (HU), maximum (HU), mean (HU), sum (HU), and SDev (HU).

### 2.6. Statistical Analyzes

The statistical significance of the collected data was assessed using the Statistical Package for Social Science (SPSS, version 17) software. To accentuate the statistical significance of the independent variables, the Kruskal–Wallis test was employed. This non-parametric statistical test is designed to ascertain whether samples originate from the same distribution. It finds application in the comparison of two or more independent samples of equal or different sample sizes. The Kruskal–Wallis test does not specify the location or extent of stochastic dominance, nor does it determine the number of pairings of groups it acquires. However, it does demonstrate that at least one sample stochastically dominates another sample. This test does not presuppose a normal distribution of the residuals because it is a nonparametric technique [37,38]. To assess the correlation between biomaterials variables at varying measurement positions, all data are expressed as the mean value and standard deviation for each group within all assessment positions. *p*-values less than 0.05 are considered statistically significant, and *p*-values less than 0.01 are regarded as highly statistically significant.

## 3. Results

**Figure 4:**
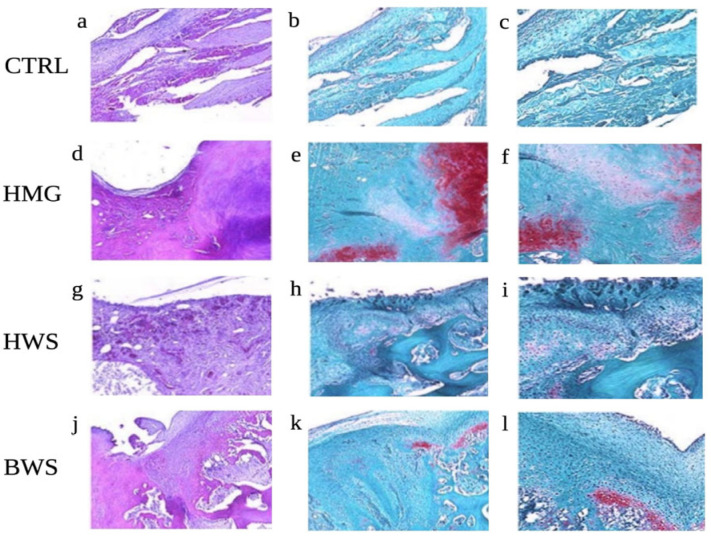
Histological evaluation of the osteochondral lesions; Representative H and E (**a**,**d**,**g**,**j**) and Safranin-O (**b**,**c**,**e**,**f**,**h**,**i**,**k**,**l**) pictures for each experimental group are shown: (**a**–**c**) CTRL; (**d**–**f**) HMG; (**g**–**i**) HWS; (**j**–**l**) BWS. Magnification: 5×: (**a**,**b**,**d**,**e**,**g**,**h**,**j**,**k**); 10×: (**c**,**f**,**i**,**l**). Several spots in the HMG samples were characterized by the presence of round, chondrocyte-like cells.

All animals included in this study survived and did not sustain any major complication post-surgery, such as inflammation of articular structure, refusal of osteochondral scaffold, negative range of motion (ROM), lameness, infection, or wound dehiscence. After a period of three months, the animals were euthanized, and the right knees were promptly subjected to a CT evaluation. Also, the samples were subsequently processed and sectioned for histological evaluation. Hematoxylin and Eosin stain displayed the absence of a valid reparative tissue in the CTRL group, Safranin O stain confirmed these data, as no GAGs were detected in this group. On the other hand, the experimental samples were characterized by the presence of a reparative tissue within the defects. In all three experimental groups, the ceramic material was still there, but it was well integrated with the healthy subchondral bone. The reparative cartilaginous layer was constituted mainly of elongated, fibrous-like cells, especially in the superficial region in all sections. However, the Safranin O stain showed some important differences between the groups: in the HWS samples, almost no GAGs were detected, suggesting that the cells invading the defects did not undergo chondrogenic differentiation. In the BWS group, positive Safranin O stain was spotted in the transition layer between the cartilaginous and the bony phase. This result suggests that an endochondral bone reparative process was active in these samples. Finally, the HMG group displayed the presence of GAGs both in the deep and superficial layer of most of the samples. Moreover, several spots in the HMG samples were characterized by the presence of round, chondrocyte-like cells. Results of histological assessment are presented in Figure 4.

**Figure 5:**
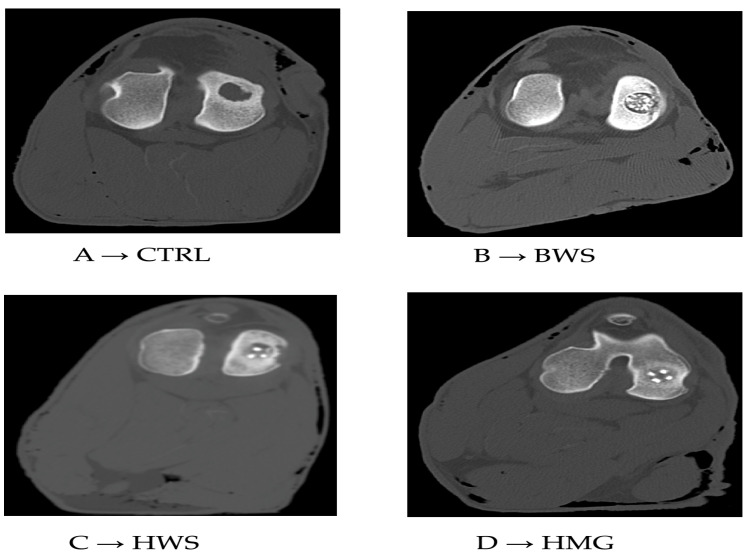
Tomographic images of respective groups. (**A**) → CT image of control group (CTRL), (**B**) → CT image of BWS, (**C**) → CT image of HWS, (**D**) → CT image of HMG scaffold.

The CT scans revealed low values of HU in ROI metrics and the absence of reparative processes in the subchondral bone of the CTRL group. The BWS group exhibited low values of HU in ROI metrics and subchondral bone integration issues. In the other groups, the presence of ceramic material (hydroxyapatite or wollastonite) was evident, but the bony phase of the scaffolds appeared to be well integrated with the surrounding subchondral bone. This observation was particularly evident in the HMG scaffold, though it was less pronounced in the HWS, as illustrated in Figure 5.

**Figure 6:**
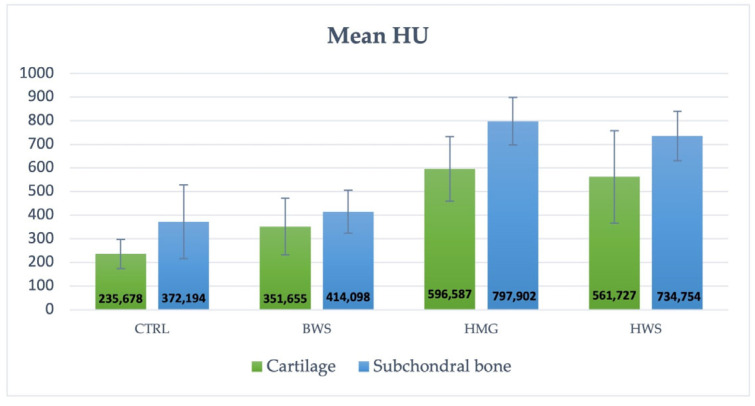
The histogram illustrates the mean value (HU) and SD of the control group and the three groups of scaffolds. The HMG scaffolds demonstrated the optimal performance for both components of osteochondral lesions, with blue indicating the cartilage portion and orange indicating the subchondral part.

In contrast, the experimental groups exhibited the presence of reparative tissue; however, its quality was found to be inadequate, particularly in the BWS group. Additionally, the integration of the HWS with the surrounding tissue appeared to be inadequate. Conversely, the HMG group exhibited a substantial reparative tissue that filled the defect; however, complete restoration of the lesion was not yet achieved. According to the standard HU scale, osteochondral structures typically range from 300 to +1000 HU [17,18,19]. In these cases, the cartilage component exhibited lower values compared to the subchondral component. This study presents the mean ROI metric value of the two components of the osteochondral structure (cartilage and subchondral bone), which fall within the reference range of Hounsfield units, as shown in Table 1. These values are also shown in Figure 6, which indicates that the best performance of scaffolds is observed in HMG biomaterial, having the highest HU values; cartilage at 596.587 HU and subchondral bone at 797.902 HU.

**Figure 7:**
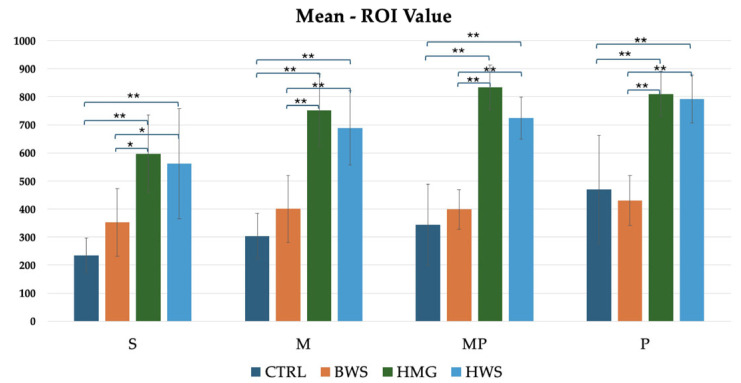
Statistically significant difference between different biomaterials in four evaluation positions inside the osteochondral part of the medial femoral condyle are indicated by symbols; ** = *p* < 0.01 (very statistically significant); * = *p* < 0.05 (statistically significant); the histogram shows comparison between all groups of mean ROI (HU) value evaluation, where there is statistical significance in all positions of evaluation.

In all the following histograms, the positions of close polygons in ROI value assessments are marked with the letters S, M, MP and P. As previously indicated in the section on “Mean ROI Value,” this is the most significant value, as it demonstrates which of the scaffolds exhibited the strongest regeneration abilities during the healing process in all evaluation positions. This is in relation to the increased tissue density, as evaluated by the HU value. As illustrated in Figure 7, the histogram reveals that HMG scaffolds exhibit the highest HU values across all evaluation positions. These are followed by HWS, with BWS and the CTRL group exhibiting similar HU values. This finding suggests that the BWS group demonstrates a reduced level of tissue regeneration. Statistical analyses revealed no statistically significant differences between the CTRL group and the BWS and HMG with HWS groups, as evidenced by the nearly identical HU values. Statistically significant differences were identified in the confrontations between the CTRL group and the HMG and HWS groups, as well as in the confrontations between the BWS group and the HMG and HWS groups. In the CTRL group’s interaction with both HMG and HWS groups, 100% of statistical significance had *p*-values less than 0.01. Similarly, in the BWS group’s interaction with both HMG and HWS groups, 75% of statistical significance had *p*-values less than 0.01, with the remaining 25% having *p*-values less than 0.05. (Figure 7).

**Figure 8:**
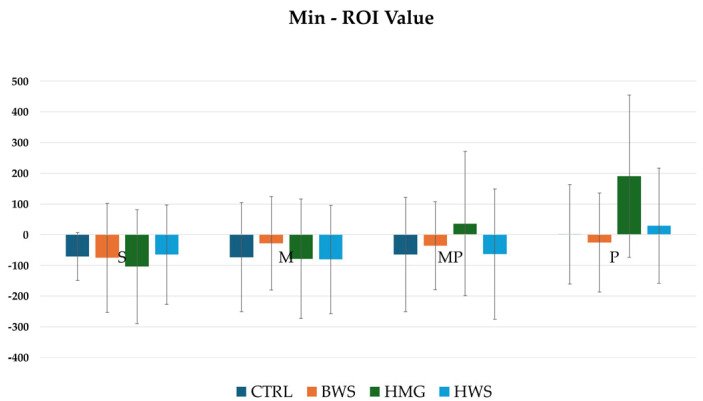
The histograms show min ROI (HU) values of different biomaterials in four evaluation positions inside the osteochondral part of the medial femoral condyle; they are not statistically significant between all groups in all evaluation positions.

Regarding the minimum ROI metric value, no statistical significance was observed between the CTRL group and the experimental groups (BWS, HMG, and HWS) in all evaluation positions. The HU values in all positions indicate that the HMG scaffolds show high average values in all positions when compared to the CTRL, BWS, and HWS groups, as shown in Figure 8.

**Figure 9:**
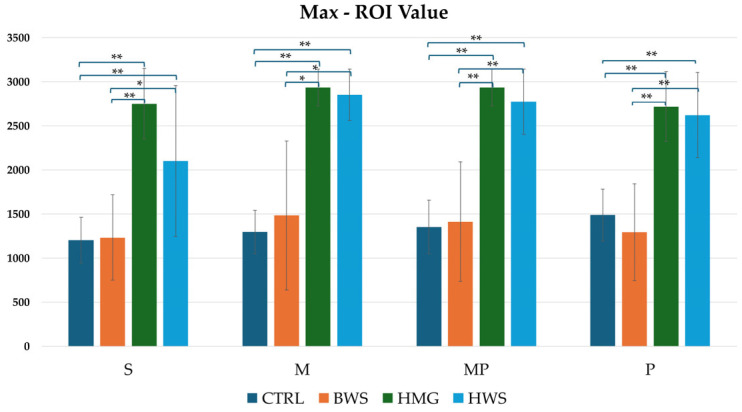
The histogram shows comparison between all groups of max ROI (HU) values in four evaluation positions inside the osteochondral part of the medial femoral condyle, where there is statistical significance in all position of evaluation. Statistically significant differences between different biomaterials are indicated by symbols; ** = *p* < 0.01; * = *p* < 0.05.

As illustrated in Figure 9, the histogram pertaining to maximum ROI values indicates that HMG scaffolds exhibit the highest HU value across all evaluation positions. These are followed by HWS, and finally by BWS and the CTRL group. Statistical analyses revealed no statistically significant differences between the CTRL group and the BWS group, or between the HMG group and the HWS group, as evidenced by the nearly identical HU values. Statistically significant differences were identified in the confrontation between the CTRL group and the HMG and HWS groups, as well as in the confrontation between the BWS group and the HMG and HWS groups. In the CTRL group’s interaction with both HMG and HWS groups, 100% of statistically significant results had *p*-values less than 0.01. Similarly, in the BWS group’s interaction with both HMG and HWS groups, 70% of statistically significant results had *p*-values less than 0.01, with the remaining 30% having *p*-values less than 0.05.

**Figure 10:**
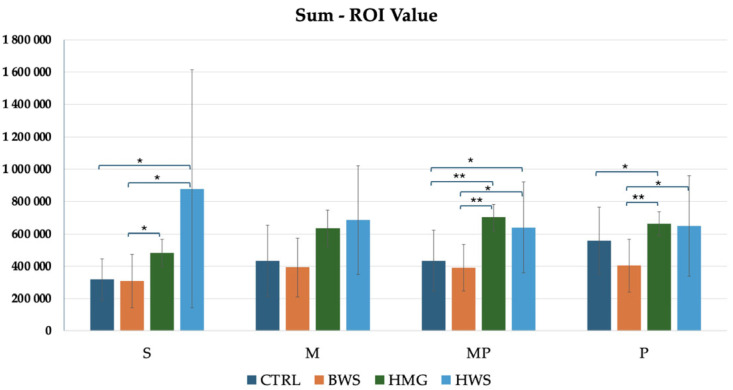
The histogram shows comparison between all groups of sum ROI (HU) values in four evaluation positions inside the osteochondral part of the medial femoral condyle, where there is statistical significance in three positions of evaluation, in the M position, there is not statistical significance between biomaterials. In S, MP, and P positions, there are statistically significant differences between different biomaterials, that are indicated by symbols; ** = *p* < 0.01; * = *p* < 0.05.

As illustrated in Figure 10, the histogram pertaining to the sum ROI values demonstrated that HMG scaffolds exhibited the highest HU values in the MP and P evaluation positions. In the S and M positions, HWS groups demonstrated the highest HU values when compared to the other groups, while the lowest HU values were observed in the BWS and CTRL groups. Statistical analyses revealed no statistically significant differences between the CTRL and BWS groups or between the HMG and HWS groups in all positions. Additionally, there was an absence of statistically significant differences between the control and experimental groups in the M position. However, statistically significant differences were observed in the confrontation between the CTRL group and the HMG and HWS groups in the S and MP positions, as well as in the confrontation between the BWS group and the HMG and HWS groups in the MP and P positions. Additionally, no statistically significant differences were observed between the CTRL and HWS groups in the P position evaluation. Conversely, no statistically significant differences were identified between the CTRL and HMG groups in the S position evaluation. The statistical analysis of the CTRL group in confrontation with the HMG and HWS groups revealed that 20% of the statistically significant results had *p*-values less than 0.01, while 80% had *p*-values less than 0.05. Similarly, the statistical analysis of the BWS group in confrontation with the HMG and HWS groups showed that 33.3% of the statistically significant results had *p*-values less than 0.01, while 66.7% had *p*-values less than 0.05.

**Figure 11:**
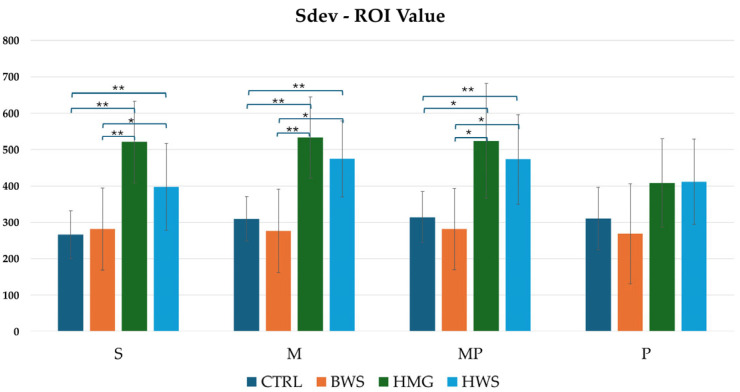
The histogram shows a comparison between all groups of Sdev-ROI (HU) values in four evaluation positions inside the osteochondral part of the medial femoral condyle, where there is statistical significance in three positions of evaluation; in the P position, there is not statistical significance between biomaterials. In S, M, and MP positions, there are statistically significant differences between different biomaterials, that are indicated by symbols; ** = *p* < 0.01; * = *p* < 0.05.

As illustrated in Figure 11, the histogram pertaining to Sdev ROI values demonstrated that HMG scaffolds exhibited the highest HU value across all evaluation positions. These were followed by HWS, and finally, BWS and the CTRL group. A statistical analysis reveals that there are no statistically significant differences between the CTRL group and the BWS and HMG with HWS groups, as evidenced by the nearly identical HU values. Additionally, no statistically significant differences are observed between the CTRL and BWS groups, or the HMG and HWS groups, in the P position evaluation. However, statistically significant differences were observed in the confrontation between the CTRL group and the HMG and HWS groups, as well as between the BWS group and the HMG and HWS groups, in the S, M, and MP positions evaluation. In the confrontation of the CTRL group with the HMG and HWS groups across all evaluation positions, 83.3% of statistically significant results had *p*-values less than 0.01, and 16.7% had *p*-values less than 0.05. Similarly, in the confrontation between the BWS group and the HMG and HWS groups, 66.7% of statistically significant results had *p*-values less than 0.01, and 33.3% had *p*-values less than 0.05.

Regarding the area (cm^2^) and length (cm) of ROI values, no statistically significant differences were observed between the study groups in all evaluation positions. These values are nearly identical across all biomaterials.

## 4. Discussion

The identification of treatments for osteochondral lesions can be significantly facilitated by research conducted on animal models. A range of animal species have been utilized in this context to identify those that most closely resemble humans in terms of anatomy and physiology. Rodents, rabbits, dogs, sheep, goats, lambs, horses, and pigs are the most frequently utilized animals in research, with sheep models demonstrating advantages in terms of the dimensions of joints, cartilage, and subchondral bone, which exhibit adequate thickness and consistency. The accessibility of joints for arthroscopic examination and the relatively limited capacity for healing in these models are further advantages. Sheep are also more cost-effective than larger animals, easier to handle, and their joint size facilitates the creation of cartilage and osteochondral defects. However, this model is not without its limitations, which include the necessity of stable facilities and adequate structures to perform surgeries and studies. These limitations permit the creation of larger lesions and the testing of various types of implants, such as scaffolds [39], bone marrow stromal cell transplants [40], and autologous fibrin preparations [41]. The objective of this study is to evaluate the regeneration of large osteochondral defects using CT scan analysis as a methodological framework, underpinned by ROI and HU values. A statistical analysis of the data obtained from the CT scan revealed that the HMG scaffolds exhibited superior performance, with statistically significant differences observed in comparison to the CTRL and BWS groups (*p*-value < 0.01). It is well established that magnesium plays a critical role in bone remodeling and has the potential to enhance the osteogenic capacity of materials based on hydroxyapatite [23,31]. This observation aligns with the enhanced outcomes observed in the HMG group. Conversely, there was a reduced integration of the HWS and BWS with the surrounding tissue. This phenomenon could be attributed to the fact that the wollastonite (WS) phase may not possess the optimal characteristics for chondrogenic development, as evidenced by the magnesium-doped hydroxyapatite in the HMG scaffold. The tomographic evaluation results indicate that the HMG scaffold demonstrates superior performance in comparison to the other groups (CTRL, BWS, and HWS), as was previously indicated in a preliminary study conducted by our research group. This article indicated that the HMG scaffold, which combines collagen and magnesium-doped hydroxyapatite, had the most promising outcomes in terms of tissue regeneration and scaffold integration. Magnesium is known to be essential to bone remodeling and may improve the osteogenic capacity of materials based on hydroxyapatite [23,31], which would explain the better results shown in the HMG group; on the other hand, there was less integration of the HWS and BWS and the surrounding tissue. A statistically significant discrepancy was identified between the CTRL and HMG groups with respect to integration and the extent of repair, as determined by histological analyses, which revealed the presence of GAGs in both the deep and superficial layers of the HMG group [3,31]. The statistical significance of the differences in the CT evaluations further supports the potential of HMG scaffolds for promoting superior osteochondral healing. Specifically, the HMG scaffolds demonstrated superior performance across a range of evaluation metrics, including integration, reparative tissue quality, and bone–cartilage regeneration. The Kruskal–Wallis test indicates that the CTRL and BWS groups exhibit similar ROI metrics, as do the HWS and HMG groups, across all evaluation positions. Additionally, no statistically significant differences were observed between the CTRL and BWS groups or the HWS and HMG groups, across all evaluation positions. In contrast, significant differences are apparent between the CTRL and HWS/HMG groups, as well as between the BWS and HWS/HMG groups. The importance of the study also underscores the fact that the HU is used in the tomographic evaluation of osteochondral defects to assess regenerative processes compared to other studies conducted in mice [42], where tomographic studies were performed to assess only the area of regeneration but not the density.

The study underscores the pivotal role of the scaffold material composition in facilitating successful osteochondral regeneration. Large osteochondral defects failed to be fully repaired during the three-month investigation period, despite the encouraging present outcomes. This observation indicates that while biomaterial scaffolds can indeed stimulate healing, further modifications or extended periods of time may be necessary to achieve complete restoration of both cartilage and subchondral bone.

Consequently, further research could center on optimizing the composition and structure of the scaffolds to enhance their regenerative properties. Specifically, the combination of bioactive molecules or growth factors with these scaffolds could potentially accelerate the differentiation of cells into the osteogenic and chondrogenic lineages, thereby enhancing the integration and restoration of the osteochondral interface. Moreover, extending the duration of the study beyond three months could yield more profound insights into the long-term efficacy of these scaffolds in promoting complete osteochondral repair. While the HMG scaffolds demonstrated superior outcomes in this study, their performance could be further enhanced by adjusting the magnesium content or exploring alternative materials that may promote both osteogenic and chondrogenic repair.

The limitations of the study are associated with the follow-up of the study evaluations, as the results were only obtained after a period of three months. The remaining limitations are associated with the subjectivity of the ROI metrics evaluations, a challenge that is mitigated to some extent by the double-blind study design. A further limitation pertains to the selection of evaluation areas, as in certain instances, sections must be discarded due to excessive reactions of the osteal and perichondral tissues.

## 5. Conclusions

The study offers significant insights into the potential of diverse biomaterials for repairing osteochondral defects in a large animal model, particularly in sheep. The results suggest that CT assessment of the HMG scaffold showed a high density of osteochondral structure compared to the other groups. While all scaffolds promoted tissue regeneration, HMG scaffolds demonstrated superior healing outcomes, characterized by the formation of more robust reparative tissue and significant defect filling. HWS also exhibited a favorable healing process, while BWS showed nearly identical values to the control group, indicating a minimal presence of regenerative tissue. A statistically significant discrepancy was identified between the treatments and the control group, with the CTRL and HMG groups exhibiting notable differences. The macroscopic and histological assessments conducted in other studies within our research group evaluated the responses of the CT examinations. These assessments revealed that HMG scaffolds exhibited high density and HU values. In comparison to our previous study, the macroscopic analysis demonstrated high integration and a substantial degree of repair. Furthermore, the histological analyses revealed the presence of GAGs in the deep and superficial layers of the scaffolds. This study underscores the significance of CT assessment as a predictive tool for evaluating hard tissue healing processes over time, as evidenced by the correlation between Hounsfield units and the macroscopic and histological assessments.

## Figures and Tables

**Table 1:** The mean value (HU) and SD (standard deviation) of the osteochondral structure is divided into two parts: cartilage and subchondral bone.

Group	Cartilage Part	Subchondral Part
CTRL	235.678 ± 61.66 HU	372.194 ± 156.27 HU
BWS	351.655 ± 120.38 HU	414.098 ± 90.37 HU
HMG	596.587 ± 136.97 HU	797.902 ± 99.63 HU
HWS	561.727 ± 196.11 HU	734.754 ± 104.65 HU

## Data Availability

The original contributions presented in this study are included in the article. Further inquiries can be directed to the corresponding author.
